# Corrigendum: Single-particle measurements of filamentous influenza virions reveal damage induced by freezing

**DOI:** 10.1099/jgv.0.001636

**Published:** 2021-08-18

**Authors:** Jack C. Hirst, Edward C. Hutchinson

**Affiliations:** ^1^​ MRC-University of Glasgow Centre for Virus Research, Sir Michael Stoker Building, Garscube Campus, 464 Bearsden Road, Glasgow G61 1QH, Scotland, UK

**Keywords:** virion

In the published version of this article, in the Methods section ‘Virion manipulations’ there was an error in the description of a sonication instrument.

The sentence ‘Sonication was performed at 50 Hz in a Kerry KC2 ultrasonic bath’ should have read ‘Sonication was performed at 38 kHz in a Kerry KC2 ultrasonic bath’.

The authors apologise for any inconvenience caused.

